# Supplementary material for: Coupling News Sentiment with Web Browsing Data Improves Prediction of Intra-Day Price Dynamics
Source: PLoS One. 2016 Jan 25;11(1):e0146576. doi: 10.1371/journal.pone.0146576 (PMC4726698; doi:10.1371/journal.pone.0146576)
Supplement: S1 Text — (PDF) [file pone.0146576.s001.pdf]

Supporting Information for "Coupling news sentiment with web browsing data improves prediction of intra-day price dynamics"

Gabriele Ranco[1,‡], Ilaria Bordino[2], Giacomo Bormetti[3,4], Guido Caldarelli[1,5,6], Fabrizio Lillo[3,4], Michele Treccani[4,7]
1IMT Institute for Advanced Studies, Piazza San Francesco 19, 55100 Lucca, Italy
2Yahoo Labs, Barcelona, Spain
3Scuola Normale Superiore, Piazza dei Cavalieri 7, 56126 Pisa, Italy
4QUANTLab, Via Pietrasantina 123, 56122 Pisa, Italy
5ISC-CNR, Via dei Taurini 19, 00185 Roma, Italy
6London Institute for Mathematical Science, South St. 35 Mayfair, London W1K 2XF, UK
7Mediobanca S.p.A, Piazzetta E. Cuccia 1, 20121 Milano, Italy
‡E-mail: gabriele.ranco@gmail.com

1 Financial time series

We consider only trading hours (i.e. from 9:30 AM to 4:00 PM) and trading days (i.e. business days at the New York Stock Exchange), and we disregard the trading events that occur out of this time window. From the financial data we create three time series: the first one is the traded volume for each minute for each company, the second one is the logarithmic returns, and the last time series is the absolute value of the logarithmic returns.

1. **Trade volume (V) time series**. It consists of the traded volume of a given company at minute time scale. We build a time series at time scale t by summing the traded volumes v_τ at the smallest time scale τ (in our case one minute). Then, the total volume $\overline{v_t}$ can be defined as follows:

$$\overline{v_t} = \sum_{\tau > t-1}^{t} v_\tau \,. \tag{1}$$

Traded volumes at intra-day time t are rescaled by a factor ζ_t^v, which is computed as the average, over all days, of the volume at time t normalized by the total daily volume. If $\overline{v_{d,t}}$ is the raw volume of day d and intra-day time t, we define the rescaled time series as

$$V_{d,t} = \frac{\overline{v_{d,t}}}{\zeta_t^v} \,, \tag{2}$$

where

$$\zeta_t^v = \frac{1}{T} \sum_{d'} \frac{\overline{v_{d',t}}}{\Lambda_{d'}} \,, \tag{3}$$

and

$$\Lambda_d = \sum_t \overline{v_{d,t}} \,, \tag{4}$$

i.e. the total volume trades on day d, and T is the total number of trading days.

2. **Price return (R) time series**. We preliminary compute the logarithmic return, defined as

$$r_t = \log_{10}(\frac{p_t}{p_{t-1}})\,, \tag{5}$$

where p_t is the last recorded price in the interval $(t-1, t]$. Returns at intra-day time t are rescaled by a factor ζ_t^r, which is computed as the average, over all days, of absolute returns at time t rescaled by the average volatility. More precisely, if $r_{d,t}$ is the raw return of day d and intra-day time t, we define the rescaled time series as

$$R_{d,t} = \frac{r_{d,t}}{\zeta_t^r}\,, \tag{6}$$

where

$$\zeta_t^r = \frac{1}{T}\sum_{d'}\frac{|r_{d',t}|}{\Xi_{d'}}\,, \tag{7}$$

and

$$\Xi_d = \mathrm{mean}(|r_{d,t}|)\,, \tag{8}$$

where we compute the mean value for all t in a day d.

3. **Volatility (σ) time series**. We employ as a simple proxy for the de-seasonalized intra-day volatility the absolute value of the rescaled return

$$\sigma_t = |R_t|\,. \tag{9}$$

Notice that the volatility σ_t is already de-seasonalized, because of the definition of R

2 Sentiment analysis

We discuss here how we tagged the news and how we performed the sentiment analysis. The news data contained in the log of Yahoo! Finance were marked with relevant stocks, identified through two different tagging methods. A first type of annotations was provided by a team of editors, who manually assessed the articles published on the Yahoo! Finance website and marked them with companies which the content of the article was relevant for. In addition to that, relevant companies were also identified by applying a proprietary entity-recognition tool. We discarded all the articles that were tagged with more than 4 companies. This threshold was chosen heuristically in a preliminary assessment, where we verified that articles tagged with 5 or more companies generally corresponds to aggregated periodic reports, which mention long lists of stocks without being really specific about any of them.

Among the obtained articles, we further processed those tagged with more than one company, introducing some additional filters to make sure we would consider the news for a company when the content of the article was really relevant to it, and not in the case where a company was only tagged due to a casual mention in the text. Such additional filters consisted in only retaining the stocks that were mentioned either in the title or in the first paragraph of the article, which typically contains a summary of the key concepts discussed in the article. Each article then contributed to the time series of all the stocks it was tagged for.

As explained in the main text, we have used *SentiStrength* adding a list of sentiment keywords of special interest and significance for the financial domain. We have tested the classification in good, bad, or neutral news using the general dictionary. In 84% of the cases the classification is the same by using both dictionary. This result indicates that our classification and the consequent analysis is pretty

robust to the choice of dictionary. Interestingly, 17% of the news classified as neutral by the general dictionary are classified as positive or negative by using the financial dictionary, while only 8% of the news classified as neutral by the financial dictionary are classified as positive or negative when using the general dictionary. This suggests that the use of a financial dictionary sharpens the capability of giving a positive or negative sign to a news.

3 Click and sentiment time series

Consistently with the financial time series, we consider only trading time and trading days. This implies that we neglect the clicks that occur out of this time window, because we are only interested in the click behaviour during the trading hours evolution of markets. From the click-through history of each news we create two time series: the first one is the total amount of clicks for each minute for each company, and the second one is the number of clicks multiplied by the sentiment score of the associated news.

1. **Click (C) time series**. Starting from the number of clicks of each news at the smallest time scale τ (in our case one minute), we build a time series at the time scale t by aggregating the number of clicks of all the news of a given company. So if we denote by N the number of news of a company, and by c^i_τ the number of clicks for news i at scale τ, the total volume $\overline{c}_t$ can be defined as

$$\overline{c_t} = \sum_{\tau > t-1}^{t} \sum_{i=1}^{N} c^i_\tau \,. \tag{10}$$

   The news that are not viewed in the time interval have zero clicks. We filter out the intra-day pattern from clicks by means of a simple methodology. Clicks at intra-day time t are rescaled by a factor ζ^c_t, which is computed as the average, over all days, of the click volume at time t normalized by the total number of daily clicks. More precisely, if $\overline{c_{d,t}}$ is the raw click volume of day d and intra-day time t, we define the time series of rescaled clicks as:

$$C_{d,t} = \frac{\overline{c}_{d,t}}{\zeta^c_t} \,, \tag{11}$$

   where

$$\zeta^c_t = \frac{1}{T} \sum_{d'} \frac{\overline{c}_{d',t}}{\Gamma_{d'}} \,, \tag{12}$$

   and

$$\Gamma_d = \sum_t \overline{c}_{d,t} \,, \tag{13}$$

   with Γ_d the total number of clicks in a day.

2. **Sentiment (S) time series**. To construct this time series we consider the sentiment of the headlines of the news. With the same notation previously used

$$S_t = \sum_{\tau > t-1}^{t} \sum_{i=1}^{N} s^i_\tau \,, \tag{14}$$

   where s^i_τ is the sign $(-1, 0, 1)$ of the sentiment of a news headline published at time τ.

Table 1. Tail exponent α and lower bound $x_{\min} > 0$ with standard errors ϵ_α and $\epsilon_{x_{\min}}$ estimated from 100 highly capitalized stocks traded in the US equity markets. Following the procedure detailed in [1], we estimate the probability distribution associated with integer numbers larger than $x_{\min}$ whose expression reads to $p(x) = x^{-1-\alpha}/\zeta(1+\alpha, x_{\min})$. The normalizing constant corresponds to the Hurwitz zeta function ζ. We report in bold the top 10 assets, as measured by the absolute number of associated news.

3. **Weighted Sentiment (WS) time series**. We multiply the click count of each news by its sentiment score. With the same notation of the click time series, we have:

$$\overline{WS}_t = \sum_{\tau > t-1}^{t} \sum_{i=1}^{N} c_\tau^i s_\tau^i \,. \tag{15}$$

This way, we weight the sign of every news by the level of attention that the news has received. We remark that for each trading day we consider all the clicks of all the news clicked in that day, not only the news that have been released in that day. Then, we define the weighted sentiment as

$$WS_t = \mathrm{sign}(\overline{WS}_t) C_t \,. \tag{16}$$

4 Estimation of power law exponents

We estimate the power law tail exponent of the distribution of the number of clicks per news by employing the R package PoweRlaw developed and maintained by Colin Gillespie, and described in [1].

5 Multiple hypotheses testing

In Tables 2 and 3 we report the results for tests of zero Spearman correlation and zero Granger causality, respectively, under the very conservative correction proposed by Bonferroni. If N_t tests are performed and the desired significance is p (in our case 5%, then only the tests with a p-value smaller than p/N_t are rejected. Since we perform 100 tests, our corrected p-value is 0.05%.

References

1. Clauset, A., Shalizi, C. R. & Newman, M. E. Power-law distributions in empirical data. *SIAM review* **51**, 661–703 (2009).

	α	ϵ_α	$x_{\min}$	$\epsilon_{x_{\min}}$		α	ϵ_α	$x_{\min}$	$\epsilon_{x_{\min}}$
AA	1.05	0.09	482	114	**JPM**	**0.99**	**0.04**	**665**	**205**
**AAPL**	**0.97**	**0.05**	**2881**	**1451**	KBH	1.33	0.20	402	98
ABT	1.39	0.33	530	261	KO	0.96	0.09	565	242
ACN	1.84	0.37	1074	313	LCC	0.95	0.08	722	371
AET	1.21	0.13	261	54	LEN	0.86	0.19	256	197
AIG	1.16	0.13	854	193	LMT	1.41	0.14	808	159
AMGN	1.60	0.33	1596	445	LNKD	0.90	0.13	735	297
AMZN	1.06	0.05	969	206	LUV	0.82	0.10	617	282
ATVI	1.47	0.23	863	192	MA	0.97	0.13	335	213
AXP	0.89	0.07	447	93	MAR	1.34	0.17	318	90
**BAC**	**0.95**	**0.05**	**807**	**200**	MCD	0.71	0.06	462	305
**BA**	**1.01**	**0.05**	**802**	**250**	M	0.86	0.09	384	200
BBY	0.74	0.13	957	526	MRK	1.53	0.20	966	213
BIIB	1.88	0.41	1081	251	MSCI	1.12	0.09	205	62
BLK	1.20	0.12	547	122	**MS**	**1.15**	**0.09**	**615**	**457**
CAT	1.14	0.11	834	224	**MSFT**	**1.06**	**0.05**	**2247**	**507**
CBS	0.82	0.07	169	75	NDAQ	1.24	0.08	213	42
**C**	**1.10**	**0.06**	**627**	**256**	NFLX	0.94	0.07	613	141
CHK	1.72	0.18	1479	215	NKE	1.09	0.14	566	143
CMCSA	1.41	0.24	1186	357	NOC	1.46	0.22	894	228
COP	1.76	0.24	1708	303	NWSA	0.89	0.06	234	117
COST	0.79	0.10	448	372	NYX	0.98	0.18	176	192
CSCO	1.32	0.11	1290	271	ORCL	0.95	0.20	523	422
CVX	1.37	0.15	782	180	PEP	0.92	0.13	565	220
DAL	1.01	0.12	959	220	PFE	1.54	0.14	1306	250
DELL	1.00	0.07	944	246	PG	1.02	0.11	809	327
DIS	0.72	0.04	283	198	QCOM	1.44	0.23	1748	363
DISH	1.05	0.17	515	391	RTN	1.83	0.47	1379	425
DOW	1.21	0.13	818	313	SBUX	0.80	0.07	453	236
DTV	1.11	0.13	438	132	S	1.09	0.14	684	424
EBAY	0.91	0.11	1612	459	TRI	0.89	0.04	122	62
**FB**	**0.95**	**0.08**	**1211**	**1077**	TSLA	0.98	0.11	3024	850
F	0.96	0.05	1188	254	TWC	1.06	0.09	403	176
FRT	1.19	0.08	412	87	TWX	0.84	0.08	260	119
GE	0.98	0.08	587	236	TXN	1.55	0.42	1060	475
GILD	1.87	0.43	1300	294	UAL	1.00	0.12	1153	659
GM	0.84	0.05	863	296	UNH	1.21	0.15	378	147
**GOOG**	**0.92**	**0.03**	**1786**	**450**	UPS	1.93	0.49	2499	721
GPS	1.28	0.17	390	158	UTX	1.49	0.20	733	209
GRPN	0.93	0.13	644	193	V	1.17	0.13	505	243
**GS**	**0.89**	**0.05**	**462**	**143**	VMW	1.50	0.45	1003	547
HAL	1.56	0.18	880	213	VOD	1.87	0.42	1850	711
HD	0.79	0.07	456	169	VZ	1.11	0.10	1001	359
HON	1.18	0.17	632	296	WAG	1.06	0.23	512	322
HPQ	1.13	0.11	786	274	WFC	0.95	0.05	874	271
IBM	1.11	0.11	1014	283	WMT	0.67	0.06	2073	517
INTC	1.35	0.32	1777	738	XOM	1.29	0.12	802	147
IT	1.32	0.13	410	98	YHOO	1.15	0.12	2546	605
JCP	0.75	0.07	2827	959	YUM	0.90	0.10	483	225
JNJ	1.51	0.19	1058	174	ZNGA	1.38	0.16	1578	338

Table 2. Percentage of companies for which we reject the null hypothesis of zero Spearman correlation at 0.05% confidence level (Bonferroni's correction).

Time interval (minutes)	$\rho(WS, R)$	$\rho(C, \sigma)$	$\rho(C, V)$
1	0	73	92
10	0	48	81
30	0	24	76
65	0	9	66
130	0	6	55

Table 3. Number of companies for which we reject the null hypothesis of no Granger causality at 0.05% confidence level (Bonferroni's correction).

Causality relation	Hourly scale
$S \to R$	1
$R \to S$	0
$R \to WS$	5
$WS \to R$	49
$V \to C$	94
$C \to V$	52
$C \to \sigma$	60
$\sigma \to C$	77
